# Subcutaneous Olanzapine at the End of Life in a Patient with Schizophrenia and Dysphagia

**DOI:** 10.1089/pmr.2020.0039

**Published:** 2020-06-11

**Authors:** Jonathan Hindmarsh, Amy Huggin, Anya Belfonte, Mark Lee, Jonathan Pickard

**Affiliations:** Specialist Centre for Palliative Care, St. Benedict's Hospice, Sunderland, United Kingdom.

**Keywords:** clozapine, hospice care, olanzapine, palliative care, schizophrenia, terminally ill

## Abstract

Currently, there is a paucity of evidence to guide the management of antipsychotic therapy at the end of life for patients with schizophrenia. A 51-year-old female with a diagnosis of palliative squamous cell carcinoma of the tonsils was admitted to her local hospice for end-of-life care. She had a history of treatment-resistant schizophrenia, which was ordinarily managed with oral clozapine and aripiprazole. Owing to a deteriorating swallow and the inappropriateness of other enteral administration routes for this patient however, it became necessary to consider alternative means by which to give essential antipsychotic medicine. A subcutaneous infusion of olanzapine was chosen as the most viable solution. During the course of the admission, her schizophrenia began to relapse with the onset of positive psychotic symptoms (paranoia and hallucinations). This was posited as likely due to interruption of her regular oral antipsychotic medication combined with insufficient olanzapine dosing. The olanzapine dose was thus subsequently titrated over the course of a week with close monitoring, and her psychotic symptoms abated. Owing to a protracted dying phase, the patient remained on subcutaneous olanzapine for a total of 56 days, which allowed for accurate assessment of her psychiatric symptoms and evaluation of therapeutic response. The findings of this case report suggest that subcutaneous olanzapine may be an appropriate alternative for patients who are unable to take their complex oral antipsychotic regimens through enteral routes at the end of life.

## Introduction

Schizophrenia is a psychotic disorder associated with chronic or recurrent psychosis, affecting up to 1% of the population.^1,2^ The disorder is debilitating, conferring a negative impact upon health, social, and economic outcomes.^2^ Schizophrenia is a syndrome and thus patients present with a collection of symptoms from various domains of psychopathology.^3^ These include positive symptoms (e.g., hallucinations and delusions), negative symptoms (e.g., blunted affect, social withdrawal, reduced expressiveness, and lack of energy), cognitive impairment (e.g., reduced attention, working memory, and executive function), and mood and anxiety disorders (e.g., depression and social anxiety disorder).^2,3^

Compared with the general population, patients with schizophrenia experience higher rates of morbidity and mortality.^1^ Indeed, schizophrenic patients have a life expectancy that is 10 to 20 years lower than average.^1^ Reasons for this are myriad, but possible contributors include reduced access to medical care, adverse effects of psychotropic medication, increased prevalence of substance abuse (mainly tobacco), reduced socioeconomic status, suicide, and increased prevalence of comorbid conditions (such as cardiovascular disease and cancer).^1,2^

Patients with schizophrenia who have other life-limiting conditions may be undertreated or even avoid treatment.^1^ A population-based study in Canada demonstrated that patients with schizophrenia were half as likely to access palliative specialists in the last six months of life compared with the general population.^4^ Such patients may express pain differently and are less likely to self-report both pain and other symptoms.^5^ Moreover, they are less likely to engage in advance care planning, despite evidence suggesting that many patients retain capacity for such decision making.^6^ Overall, patients with schizophrenia are at a greater risk of receiving poorer end-of-life care than other patient demographics.^2^

Antipsychotics are used to manage the positive symptoms of schizophrenia, and for many patients they are a life-long treatment. Such agents are less effective at managing negative symptoms.^7^ There is a dearth of evidence guiding the optimization of antipsychotic therapy in patients with life-limiting illnesses, particularly during the end-of-life period when oral access may be lost. Clinicians should be cautious about deprescribing antipsychotics, as positive psychiatric symptoms can worsen as a consequence. Furthermore, the stress of the underlying medical illness may also exacerbate psychiatric symptoms.^2^

We present a case of a 51-year-old female with treatment-resistant schizophrenia who presented with conservatively managed squamous cell carcinoma (SCC) of the tonsils. The trajectory of her illness was such that she experienced a protracted dying phase, characterized by fatigue, fluctuating wakefulness, and a diminishing and variable oral intake—a consequent impediment to the consistent administration of critical medications.

## Case Description

A 51-year-old female with a diagnosis of treatment-resistant schizophrenia was admitted to her local hospice. Historically her schizophrenia had been resistant to a number of antipsychotic agents. She had been functioning relatively independently in a supported living center for five years and, from a psychiatric viewpoint, had remained stable on her oral medication regimen, which consisted of aripiprazole 10 mg daily and clozapine 50 mg in the morning and 125 mg at night.

She had been diagnosed with SCC of both tonsils and uvula (T3 N3 M0), which was being conservatively managed. The N3 node was located in the left mandibular region and presented as a large painful mass causing a pressure effect on local structures. Upon admission to her local hospice, it was evident that her swallow was deteriorating due to the local effects of her cancer and that consistent administration of oral antipsychotic agents could not be sustained.

The patient had capacity to make the majority of decisions regarding her care. She had a supportive family who liaised with the medical team regularly and were invaluable in establishing the patient's typical behaviors and mental health status. The placement of an enteral feeding tube (both percutaneous endoscopic gastrostomy and nasogastric tube) and the possibility of total parenteral nutrition were discussed, but it was agreed by all not to proceed with such interventions due to the risks of harm, lack of long-term benefit (especially since no active or palliative treatment options were available for her SCC), and the distress it would likely cause the patient. Despite a deteriorating swallow, the patient was able to intermittently consume ice cream and nutritional supplements but had been eating limited amounts, even before the deterioration in her swallow, due to anorexia–cachexia syndrome—an archetypal and familiar phenotype of advanced malignant disease.^8^

Three weeks after admission, as a consequence of both the local and systemic effects of her advancing cancer, the patient's swallow had deteriorated to the point wherein consistent oral administration of her antipsychotics was no longer viable. Having excluded other enteral routes, we sought a suitable parenteral alternative. Continuation of antipsychotics was of particular importance, as her history of treatment-resistant schizophrenia and expected prognosis extending into several weeks meant she was at high risk of relapse.

We considered administering both long- and short-acting intramuscular antipsychotic agents. The patient, however, was averse to receiving injections that could be painful. On multiple occasions throughout the admission, the patient had refused the placement of an intravenous cannula, and had refused venipuncture for blood sampling, as she found these procedures both painful and prohibitively distressing. This, combined with reduced muscle mass as a consequence of anorexia–cachexia syndrome, meant that intramuscular administration would not be a viable solution, particularly for repeated injections. The use of long-acting injectable (LAI) antipsychotics also presented additional challenges. If adverse effects to an LAI antipsychotic occurred, it would not be possible to withdraw the medication, and ongoing adverse effects would have had the potential to significantly compromise quality of life. In addition, LAI antipsychotic agents, compared with standard release products, do not allow for rapid dose titration or de-escalation in response to changes in clinical condition and therapeutic response. The ability to optimize medications promptly at the end of life is essential to be able to maximize therapeutic efficacy and minimize adverse effects, to ultimately improve quality of life in situations wherein time may be short. Lastly, experience regarding rotation from oral clozapine and aripirazole to an LAI antipsychotic is lacking. With all other viable routes exhausted, the team was pleased to find that the patient fortunately did not find subcutaneous injections painful, and so would contentedly receive medications through this route.

Consequently we opted for a standard-release antipsychotic agent that could be administered subcutaneously. Previous exposure to a phenothiazine antipsychotic had caused severe muscle spasms. Thus subcutaneous levomepromazine was avoided and haloperidol had previously been ineffective. Subcutaneous olanzapine was thus chosen and trialed initially at a dose of 10 mg once a day. This dose was selected based upon manufacturer recommendations^8^ and supported by a systematic review of 10 studies that demonstrated that negligible further improvement in psychotic symptoms was conferred when doses surpassed 10 mg per day.^9^

For four weeks, the patient was able to continue interacting with her family and the ward team, without resurgence of psychotic symptoms. Four weeks after commencing the olanzapine however, her family noticed that she was exhibiting signs that they identified as relapse of her schizophrenia. She had become more withdrawn, distressed, and suspicious of her family and the staff. Her mental state was carefully assessed and, after the exclusion of all other reversible causes, her symptoms were attributed to an exacerbation of her existing psychiatric disorder rather than a new delirium. Therapeutic drug monitoring for olanzapine was not performed, as the patient had declined any further blood sampling. Consequently, olanzapine was carefully titrated according to clinical response and tolerability up to a dose of 10 mg twice a day in a stepwise manner over the course of a week, and all of her psychiatric symptoms resolved.

The patient remained on olanzapine 10 mg twice daily, as subcutaneous bolus injections for a further eight days. Over the following days, however, she began spending more of her time in bed and sleeping for longer periods. We considered if this may have been an adverse effect of the olanzapine, yet the multidisciplinary team assessed that her rate of deterioration, diminishing appetite, and significant weight loss to be more congruous with the expected disease trajectory of a patient dying from progressive cancer. To minimize disturbances, avoid recurrent bolus injections, and further simplify the regimen, we switched to administering olanzapine 20 mg for 24 hours as a continuous subcutaneous infusion through a syringe driver.

The patient died peacefully 14 days later with her family by her side. Over the course of the admission, olanzapine was administered subcutaneously for a total of 56 days. Administration of olanzapine in this manner was well tolerated with no injection site reactions or systemic toxic effects being observed or reported by the patient or her family. These findings suggest that olanzapine at a dose of 20 mg per day was both efficacious and well tolerated in the management of her treatment-resistant schizophrenia in a palliative care setting. The resolution of her paranoia allowed the patient to interact with her family, thus improving her quality of life. No trigger was ever identified for her deterioration in mental health, but it was felt to be a combination of disease progression, pain, and interruption to her usual antipsychotic regimen.

## Discussion

In imminently dying patients, the deprescribing of antipsychotics may be uneventful. However, with a protracted dying phase, relapse of positive symptoms becomes a more prominent consideration. Locally there are anecdotal reports of clinicians continuing antipsychotic therapy (often subcutaneously) during the dying phase, for patients deemed at high risk of recurrence of psychotic symptoms. An absence of a parenteral formulation of clozapine, coupled with an indication confining its use to those patients with symptoms refractory to sequential trials of two different antipsychotic agents,^7^ provides further challenges in maintaining therapeutic efficacy at the end of life.

Olanzapine is an atypical antipsychotic with a broad spectrum of activity, similar to that of clozapine, antagonizing D_1_, D_2_, D_3_, D_4_, 5HT_2A_, 5HT_2C_, 5HT_3_, 5HT_6_, 5HT_7_, adrenergic (α_1_ and α_2_), histaminergic (H_1_), and muscarinic receptors.^8^ Although there are reports of olanzapine being administered subcutaneously for indications other than schizophrenia, this is off-label.

Elsayem et al.^10^ performed a small (*n* = 24) prospective open-label study in patients with advanced cancer and agitated delirium. Patients who failed to respond to haloperidol ≥10 mg per day were given subcutaneous olanzapine 5 mg *tid*, increasing to 10 mg *tid* if additional rescue haloperidol doses were required. Olanzapine was well tolerated, with potential systemic adverse effects observed in four patients. Noteworthy in this study was the fact that some patients were given olanzapine 10 mg *tid*, which is beyond the recommended maximum daily dose of 20 mg, and moreover all patients could receive rescue doses of haloperidol.^8^ Therefore, it is not known whether adverse effects occurred as a result of combined antipsychotics at doses above recommended levels. In total, 167 individual subcutaneous injections were given and no injection site reactions were observed. Overall, it would appear that subcutaneous olanzapine is well tolerated.

Structural similarities between clozapine and olanzapine means the latter may be posited as an appropriate substitute for the former^10^—an observation supported by the literature: a randomized double-blind parallel study^11^ (*n* = 147) and a double-blind noninferiority study^12^ (*n* = 190) both showed olanzapine had efficacy similar to clozapine, in reducing positive and negative symptom burden, in those with treatment-resistant schizophrenia. Furthermore, a small study (*n* = 20)^13^ cross-tapered clozapine responders to olanzapine and demonstrated equivalent efficacy in 90% of patients. With efficacy comparable with clozapine and proven tolerability in its subcutaneous form, olanzapine became the most suitable choice for management of our patient's positive symptoms.

In conclusion, this case report highlights that patients with schizophrenia at the end of life are at risk of relapsing psychotic symptoms, especially when the dying phase is protracted and enteral administration of established antipsychotic therapy is no longer possible. Prompt management of such symptoms in a palliative care setting is essential to improve quality of life. The use of subcutaneous olanzapine appears to be a safe, tolerable, and effective way of managing patients with treatment-resistant schizophrenia at the end of life when swallow or alternative enteral routes are compromised, inappropriate, or not possible. Nevertheless, these observations need to be confirmed in a larger cohort of patients.

## Consent

The patient provided verbal consent. Written consent was provided by their family.

## Abbreviations Used

LAI: long-acting injectable
SCC: squamous cell carcinoma
